# Smoking induces expression of ligands of the immune receptor NKG2D

**DOI:** 10.1186/ar3659

**Published:** 2012-02-09

**Authors:** Caroline Ospelt, Giovanni G Camici, Fabia Brentano, Peter Künzler, Christoph Kolling, Renate E Gay, Beat A Michel, Steffen Gay

**Affiliations:** 1 Center of Experimental Rheumatology and Center of Integrative Human Physiology (ZIHP), University Hospital, Zurich, Switzerland; 2 Institute of Physiology, University of Zurich, Switzerland; 3 Schulthess Clinic, Zurich, Switzerland

## Background

While different studies confirmed an increased risk for smokers to develop rheumatoid arthritis (RA), the mechanisms behind this phenomenon are not known up to now. In all probability, smoking induces expression or post-translational modification of immune activating proteins which then initiate an autoimmune reaction in individuals with a susceptible genetic background. To identify these triggering molecules we screened joints of mice that were exposed to cigarette smoke for differences of gene expression and verified our results in synovial tissues of human smokers.

## Methods

C57BL/6 mice were exposed to cigarette smoke (n = 6) or room air (n = 8) in a whole body exposure chamber for 3 weeks. Protein and mRNA was isolated from murine ankle joints and from synovial tissues obtained from smoking (n = 4) and non smoking (n = 5) RA patients undergoing joint replacement surgery. Tissues were further analysed by Affymetrix microarrays, Real-time PCR or immunoblotting.

## Results

Since data from microarray experiments had shown increased levels of the immune receptor NKG2D ligand histocompatibility 60 (H60) after cigarette smoke exposure, we measured H60 expression levels by Real-time PCR in ankle joints of smoke exposed and control mice. H60 transcript levels were 3.2 fold higher in joints of smoke-exposed mice compared to control mice (dCT 12.5 +/-0.3 versus 14.2 +/-0.4, p = 0.03). Upregulation of H60 protein after smoke exposure was also seen in immunoblotting experiments. Since H60 is not expressed in humans, we analysed expression of the 7 human NKG2D ligands RAET1E, RAET1G, MICA, MICB, and ULBP1-3 in synovial tissues of RA patients. Transcripts of ULBP1-3 were not detectable in synovial tissues and there was no difference in the expression levels of RAET1G and RAET1E in synovial tissues of smokers compared to non smokers. However, expression levels of MICA and MICB were 2.3 and 2.8 fold higher in synovial tissues of smokers than in non-smokers (dCT 11.3 +/-0.2 versus 10.1 +/-0.3, p = 0.03 and dCT 10.8 +/-0.3 versus 9.3 +/-0.5, p = 0.03).

## Conclusion

We found that smoking induces the expression of ligands of the activating immune receptor NKG2D in murine as well as in human joints. Since dysregulated expression of NKG2D ligands has been previously implicated in induction of autoimmune responses, continuous excess of NKG2D ligands in joints of smokers might be a trigger for the development of RA in susceptible individuals.

